# The fusion gene *LRP1*–*SNRNP25* drives invasion and migration by activating the pJNK/37LRP/MMP2 signaling pathway in osteosarcoma

**DOI:** 10.1038/s41420-024-01962-z

**Published:** 2024-04-27

**Authors:** Peipei Xing, Haotian Liu, Wanyi Xiao, Gengpu Zhang, Chao Zhang, Zhichao Liao, Ting Li, Jilong Yang

**Affiliations:** 1 Department of Bone and Soft Tissue Tumor, Tianjin Medical University Cancer Institute & Hospital, Tianjin, 300060 PR China; 2 National Clinical Research Center for Cancer, Key Laboratory of Cancer Prevention and Therapy, Tianjin’s Clinical Research Center for Cancer, Tianjin Medical University Cancer Institute & Hospital, Tianjin, 300060 PR China; 3 Radiation Oncology Department, Tianjin Medical University General Hospital, Tianjin, 300052 PR China

**Keywords:** Sarcoma, Bone cancer

## Abstract

Through transcriptome sequencing, we previously identified a new osteosarcoma-specific, frequent fusion gene, *LRP1*–*SNRNP25*, and found that it played an important role in tumor cell invasion and migration. However, the specific mechanism remains unclear. In this article, whole-genome sequencing further confirmed that the *LRP1*–*SNRNP25* fusion gene is formed by fusion of *LRP1* exon 8 and *SNRNP25* exon 2. In vitro, scratch and Transwell assays demonstrated that the migration and invasion abilities of *LRP1–SNRNP25*-overexpressing osteosarcoma cells were significantly increased. To explore the molecular mechanism of the *LRP1–SNRNP25* fusion in affecting osteosarcoma cell migration and invasion, we evaluated the migration and invasion-related molecular signaling pathways by western blotting. Some migration- and invasion-related genes, including pJNK and MMP2, were upregulated. Coimmunoprecipitation–mass spectrometry showed that 37LRP can interact with pJNK. Western blotting confirmed that LRP1–SNRNP25 overexpression upregulates 37LRP protein expression. Immunofluorescence staining showed the intracellular colocalization of LRP1–SNRNP25 with pJNK and 37LRP proteins and that LRP1–SNRNP25 expression increased the pJNK and 37LRP levels. Coimmunoprecipitation (co-IP) confirmed that LRP1–SNRNP25 interacted with pJNK and 37LRP proteins. The pJNK inhibitor SP600125 dose-dependently decreased the pJNK/37LRP/MMP2 levels. After siRNA-mediated 37LRP knockdown, the MMP2 protein level decreased. These two experiments proved the upstream/downstream relationship among pJNK, 37LRP, and MMP2, with pJNK the farthest upstream and MMP2 the farthest downstream. These results proved that the *LRP1*–*SNRNP25* fusion gene exerts biological effects through the pJNK/37LRP/MMP2 signaling pathway. In vivo, *LRP1*–*SNRNP25* promoted osteosarcoma cell growth. Tumor growth was significantly inhibited after SP600125 treatment. Immunohistochemical analysis showed that the pJNK, MMP2, and Ki-67 protein levels were significantly increased in tumor tissues of *LRP1*–*SNRNP25*-overexpressing cell-injected nude mice. Furthermore, lung and liver metastasis were more prevalent in these mice. In a word, *LRP1–SNRNP25* promotes invasion, migration, and metastasis via pJNK/37LRP/MMP2 pathway. *LRP1*–*SNRNP25* is a potential therapeutic target for *LRP1*–*SNRNP25*-positive osteosarcoma.

## Introduction

Osteosarcoma occurs most frequently in children and adolescents and is characterized by frequent recurrence and metastasis [1, 2]. As a malignant tumor with a highly unstable genome, osteosarcoma exhibits variations in chromosome number and structure, resulting in amplification or deletion mutations in some oncogenes and tumor suppressor genes, for example, amplification of MDM2, MYC, and RUNX2 and deletion of TP53 and RB1 [3–5]. Amplification or deletion of these genes is thought to play an important role in the development of osteosarcoma. Among these mutations, the frequency of TP53 and RB1 mutations is as high as 30–40%, and deletion of the WWOX tumor suppressor gene has recently been widely studied. Therefore, studying the effects of gene amplification or deletion on osteosarcoma development and prognosis at the gene level is a highly important research direction.

Fusion genes are new chimeric genes formed by the fusion of more than two gene coding regions that are regulated by the same sequence; mechanistically, fusion genes may be formed through chromosome translocation or chromosome inversion [6]. In some tumors, fusion genes have become recognized as a key factor in tumorigenesis, for example, *BCR*–*ABL1* in chronic myelogenous leukemia, *EWSR*–*FLI-1* in Ewing sarcoma, *SYT*–*SSX* in synovial sarcoma, *NPM*–*ALK* in lymphoma, *ETV6*–*NTRK3* in breast cancer, *TMPRSS2*–*ETS* in prostate cancer, and *EML4*–*ALK* in lung cancer [6–11]. In recent years, an increasing number of fusion genes have been discovered in tumors, and considerable progress has been made in diagnosis and treatment. However, in osteosarcoma, only two fusion genes have been reported: Debelenko et al. reported the discovery of a single *EWSR1*–*CREB3L1* fusion gene in a case of small-cell osteosarcoma, and Kang et al. reported *Rab22a*–*NeoF1* [12–14].

In our previous study, we successfully identified and reported a new fusion gene, *LRP1*–*SNRNP25*, through whole-transcriptome sequencing of samples from 11 untreated osteosarcoma patients and verified its role in promoting the invasion of SAOS2 osteosarcoma cells in vitro [15]. However, the mechanism by which *LRP1*–*SNRNP25* promotes the invasion and migration of osteosarcoma cells remains unclear. In this study, we validated the existence and structure of the fusion gene *LRP1*–*SNRNP25* at the DNA level by whole-genome sequencing (WGS). Our in vitro results showed that the fusion gene *LRP1*–*SNRNP25* promotes the invasion and migration of osteosarcoma cells by increasing the pJNK/37-kDa laminin receptor precursor (37LRP)/MMP2 protein levels. In vivo, *LRP1*–*SNRNP25* promoted the growth of osteosarcoma cells, and the pJNK inhibitor SP600125 significantly inhibited tumor growth induced by *LRP1*–*SNRNP25* overexpression. On the other hand, osteosarcoma cells overexpressing *LRP1–SNRNP25* were more likely to undergo lung and liver metastasis. Our results provide the foundation for targeted therapy of patients with *LRP1*–*SNRNP25* fusion-positive osteosarcoma.

## Results

### Verification of the existence and structure of *LRP1*–*SNRNP25* at the DNA level by WGS

In a previous study, the results of transcriptome sequencing showed that the potential breakpoints resulting in the formation of the fusion gene *LRP1*–*SNRNP25* were located in exon 8 of *LRP1* and exon 2 of *SNRNP25*. However, no evidence was provided at the DNA level for the structure of *LRP1*–*SNRNP25*. To close this knowledge gap and further confirm the mechanism of *LRP1*–*SNRNP25* formation, the HiSeq X Ten platform was used for WGS of an *LRP1*–*SNRNP25*-positive sample to further explore the structure of the fusion gene.

**Fig. 1:**
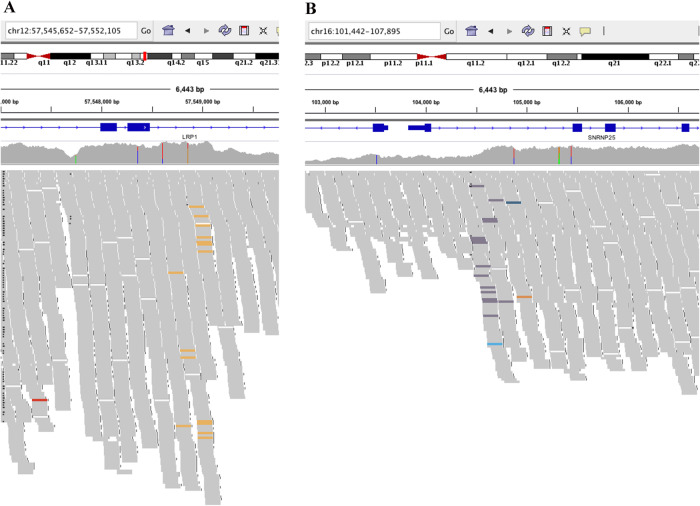
WGS verified the existence and structure of LRP1–SNRNP25 at the DNA level and confirmed that fusion rearrangement occurred through a balanced translocation between exon 8 of LRP1 and exon 2 of SNRNP25. **A** Abnormality in exon 8 of the LRP1 gene; **B** abnormality in exon 2 of the SNRNP25 gene.

We extracted genomic DNA from *LRP1*–*SNRNP25*-positive osteosarcoma samples, subjected the samples to WGS on the HiSeq X Ten platform at Huada Gene, and obtained raw data (HiSeq X Ten: Q30 ≥ 75%, sequencing strategy: PE150). The results confirmed the presence of a chromosomal rearrangement and showed that the *LRP1*–*SNRNP25* fusion gene is formed by the fusion of exon 8 of *LRP1* and exon 2 of *SNRNP25* (Fig. 1).

### The fusion gene *LRP1*–*SNRNP25* can significantly promote the migration and invasion of osteosarcoma cells

**Fig. 2:**
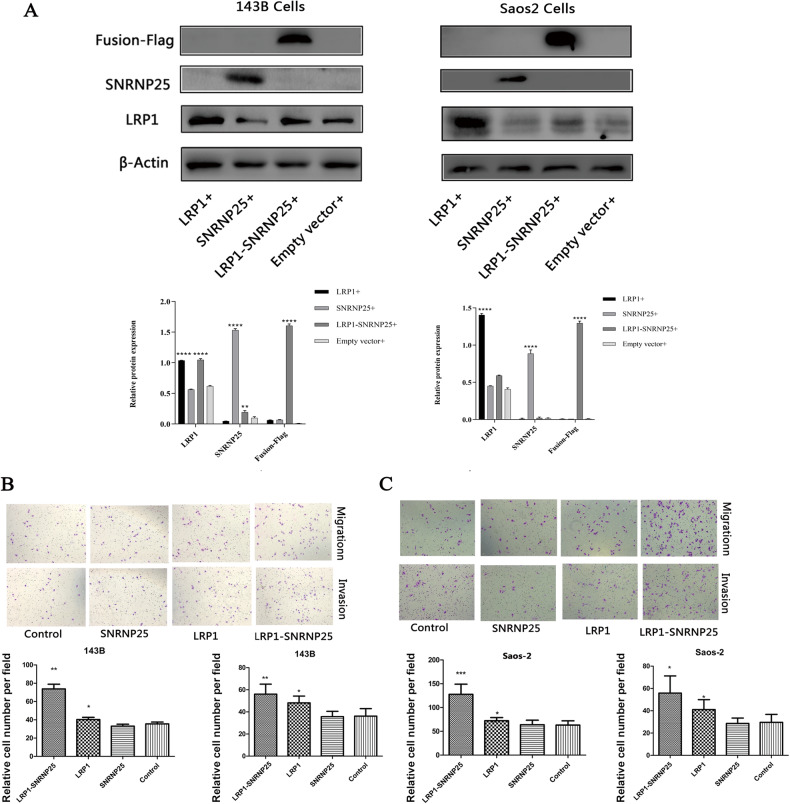
*LRP1*–*SNRNP25* promotes osteosarcoma cell invasion and migration in vitro. **A** Western blot analysis confirmed the expression of *LRP1*–*SNRNP25, LRP1*, and *SNRNP25* in 143B and SAOS2 cells. **B** The *LRP1*–*SNRNP25* fusion promoted the migration and invasion of 143B cells. **C** The *LRP1*–*SNRNP25* fusion promoted the migration and invasion of SAOS2 cells. Statistical analyses are shown below the corresponding graph. All data were analyzed by a *t* test. *P < 0.05, **P < 0.01, ***P < 0.001, and ****P < 0.0001.

First, we constructed SAOS2 and 143B osteosarcoma cell lines that stably overexpressed *LRP1–SNRNP25*, *LRP1*, *SNRNP25*, or the corresponding empty vector. The *LRP1–SNRNP25*, *LRP1*, *SNRNP25*, or empty vector was transfected into osteosarcoma cells with Lipo3000, and the cells were cultured for 48 h. Then, G418 was added to the culture medium of the transfected cells; the best concentration of G418 for screening of SAOS2 cells was 600 μg/ml, and the best concentration of G418 for screening of 143B cells was 500 μg/ml. Next, both the SAOS2 and 143B osteosarcoma cell lines were retransfected, and a nontransfected group was also established. After 48 h, the cells were cultured with the best screening concentration of G418 until most of the cells in the control group were no longer viable, and culture was then continued for 2 weeks until the formation of a single-cell clone. At this point, the stable cell line had been generated. Next, we measured the expression levels of *LRP1*–*SNRNP25*, *LRP1*, and *SNRNP25* by western blotting. As mentioned in the “Materials and Methods” section, because the vector used to construct the *LRP1*–*SNRNP25* expression plasmid contained a DDK (flag) tag, an anti-flag antibody was used to evaluate the expression of *LRP1*–*SNRNP25*. The expression of LRP1, SNRNP25, and the LRP1–SNRNP25 fusion protein was increased compared with that in the empty vector group, proving that the osteosarcoma cell lines SAOS2 and 143B expressing *LRP1*, *SNRNP25*, and *LRP1*–*SNRNP25* were successfully generated and could be used for subsequent experiments in vitro (Fig. 2A).

Then, in the scratch assay, we found that in both 143B and SAOS2 cells, the wound width in the *LRP1*–*SNRNP25*-overexpressing cell group was significantly lower than that in the *LRP1*, *SNRNP25*, and empty vector cell groups, suggesting that the fusion gene *LRP1*–*SNRNP25* can significantly promote the migration of osteosarcoma cells (Fig. 2B, C and Supplementary Fig. 1).

Next, we performed Transwell assays, which further confirmed that overexpression of the *LRP1*–*SNRNP25* fusion gene significantly promoted the invasion and migration of SAOS2 and 143B cells (Fig. 2B, C).

### *LRP1*–*SNRNP25* promotes osteosarcoma cell invasion and migration by upregulating pJNK and MMP2 expression

**Fig. 3:**
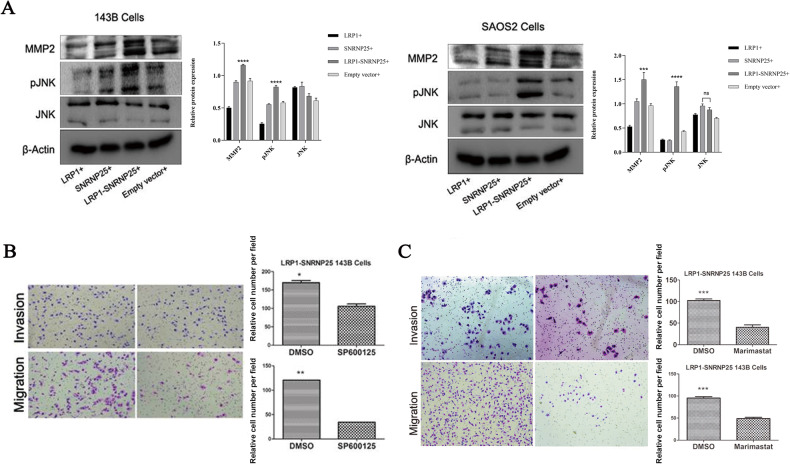
Protein levels of pJNK and MMP2 in LRP1–SNRNP25-overexpressing cells and treatment with the corresponding inhibitors. **A** Western blot analysis showed that *LRP1*–*SNRNP25* overexpression significantly increased the protein levels of pJNK and MMP2 compared with those in the other groups. **B** The pJNK inhibitor SP600125 significantly inhibited the invasion and migration of *LRP1*–*SNRNP25*-overexpressing 143B cells. **C** The MMP2 inhibitor marimastat significantly inhibited the invasion and migration of *LRP1*–*SNRNP25*-overexpressing 143B cells.

To explore the specific molecular mechanism by which *LRP1*–*SNRNP25* promotes the invasion and migration of osteosarcoma cells, we first examined the levels of proteins involved in invasion and migration, such as ERK/pERK and JNK/pJNK in the MAPK signaling pathway and Akt/pAkt in the PI3K/Akt signaling pathway, and cytoskeleton-, cell movement-, and cell adhesion-related proteins, such as FAK, MMPs, and Rac1, and the RhoA, by western blotting. *LRP1*–*SNRNP25* overexpression significantly increased the protein levels of pJNK and MMP2 (Fig. 3A), while the protein levels of JNK, ERK/pERK, Akt/pAkt, FAK/pFAK, MMP9, Rac1, and RhoA were not significantly changed (Supplementary Fig. 2). Therefore, pJNK and MMP2 may function as signaling molecules downstream of *LRP1*–*SNRNP25* to influence the biological behavior of osteosarcoma cells.

Next, we used the pJNK inhibitor SP600125 to reduce the level of pJNK in *LRP1*–*SNRNP25*-overexpressing 143B cells and found that this reduction significantly decreased the ability of *LRP1*–*SNRNP25* to promote the invasion of 143B cells (Fig. 3B). We then used the MMP2 inhibitor marimastat to inhibit MMP2 expression in *LRP1*–*SNRNP25*-overexpressing 143B cells and found that inhibition of MMP2 significantly decreased the ability of *LRP1*–*SNRNP25* to promote the invasion of 143B cells (Fig. 3C). These results suggest that *LRP1*–*SNRNP25* may promote the invasion and migration of osteosarcoma cells by increasing the protein levels of pJNK and MMP2.

### *LRP1*–*SNRNP25* upregulates MMP2 expression through the interaction between pJNK and 37LRP

**Fig. 4:**
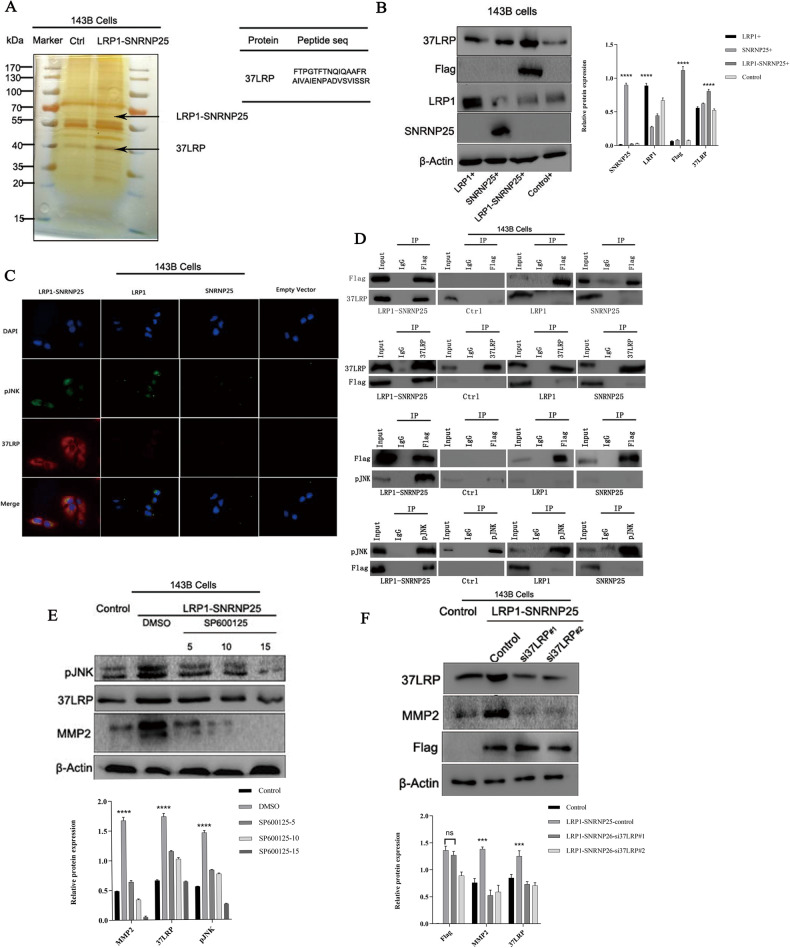
The *LRP1*–*SNRNP25* fusion upregulates MMP2 expression through the interaction between pJNK and 37LRP. **A** IP–MS identified 37LRP, which interacts with LRP1*–*SNRNP25. **B** Western blot analysis showed that *LRP1*–*SNRNP25* increased the protein expression level of 37LRP. **C** Immunofluorescence staining revealed that LRP1–SNRNP25, pJNK, and 37LRP were colocalized and that LRP1–SNRNP25 increased the protein levels of pJNK and 37LRP. **D** Co-IP also confirmed the interaction among LRP1–SNRNP25, pJNK, and 37LRP. **E** After the reduction of the pJNK level by SP600125 treatment, the expression of 37LRP and MMP2 was decreased. **F** After the knockdown of 37LRP, the expression of MMP2 was decreased.

To further investigate the proteins that interact with the fusion protein *LRP1*–*SNRNP25*, we performed co-IP and mass spectrometry. In 143B cells, based on the results of mass spectrometry, we screened the protein 37LRP with a high mass spectrometry score and matching peptide sequence (Fig. 4A). Previous studies have reported that 37LRP plays an important role in tumor invasion and metastasis and that 37LRP is regulated by pJNK and can promote the secretion of many proteolytic enzymes, including MMP2, by binding to laminin to change its conformation [16–18]. Therefore, we hypothesized that in osteosarcoma cells, 37LRP might be involved in the processes of invasion and migration induced by the *LRP1*–*SNRNP25* fusion.

To explore the associations of 37LRP with LRP1–SNRNP25, pJNK, and MMP2, we used western blot analysis, co-IP, and immunofluorescence staining. In 143B cells, western blot analysis showed that LRP1–SNRNP25 overexpression increased 37LRP protein expression (Fig. 4B). Immunofluorescence staining revealed and confirmed that LRP1–SNRNP25, pJNK, and 37LRP were colocalized in the cytoplasm and that overexpression of *LRP1*–*SNRNP25* resulted in increased protein levels of pJNK and 37LRP (Fig. 4C). Next, we investigated protein–protein interactions by co-IP. In 143B cells overexpressing *LRP1*–*SNRNP25*, *LRP1*, *SNRNP25*, and empty vector, we first used an anti-flag antibody to recognize the pJNK and 37LRP proteins and found that this antibody could indeed interact with the pJNK and 37LRP proteins. Then, we used anti-pJNK and anti-37LRP antibodies to recognize the flag-tagged proteins and obtained the same results, indicating that the LRP1–SNRNP25 fusion protein can interact with the pJNK and 37LRP proteins (Fig. 4D). These results suggest that the LRP1–SNRNP25 protein might regulate the pJNK and 37LRP protein levels.

Next, to further demonstrate the regulation of 37LRP by pJNK in osteosarcoma cells, we treated *LRP1*–*SNRNP25*-overexpressing osteosarcoma cells with the pJNK inhibitor SP600125 (5, 10, and 15 μM) and treated the control group with the same concentration of DMSO. The expression of 37LRP was inhibited after SP600125 reduced the level of pJNK, and the higher the concentration of SP600125, the lower the level of pJNK (Fig. 4E). This finding proves that 37LRP is downstream of pJNK and is regulated by pJNK. In addition, MMP2 expression also decreased with the decrease in the 37LRP expression level.

Finally, to further confirm the regulatory relationship between 37LRP and MMP2, we used two siRNAs targeting 37LRP to knockdown 37LRP expression and found that the expression level of MMP2 was decreased in *LRP1*–*SNRNP25*-overexpressing cells (Fig. 4F). This result indicated that MMP2 is the downstream signaling effector of 37LRP, consistent with previously published data.

These results suggest that the *LRP1*–*SNRNP25* fusion gene affects the biological behavior of osteosarcoma cells through the downstream pJNK/37LRP/MMP2 signaling pathway.

### *LRP1*–*SNRNP25* promotes tumor progression in vivo

**Fig. 5:**
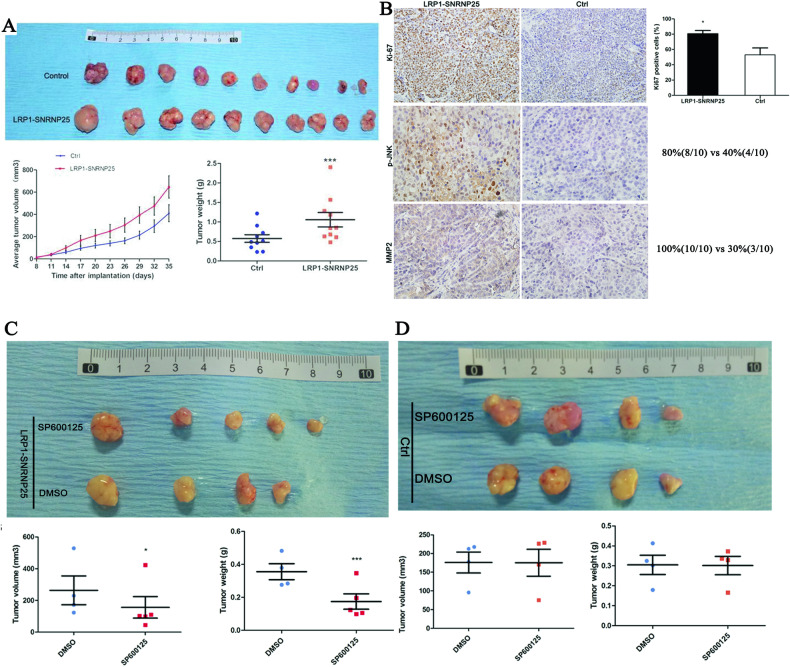
The *LRP1*–*SNRNP25* fusion promotes tumor growth. **A** The *LRP1*–*SNRNP25* fusion promoted the growth of 143B xenografts. **B** Comparison of pJNK, MMP2, and Ki-67 protein levels between the *LRP1*–*SNRNP25* overexpression group and control group. **C** SP600125 inhibited tumor growth in the *LRP1*–*SNRNP25* overexpression group. **D** SP600125 had no significant effect on tumor growth in the control group.

In the in vivo experiments, we first divided the nude mice into control group and *LRP1*–*SNRNP25* overexpression group, with ten mice in each group. We found that xenograft tumors overexpressing *LRP1*–*SNRNP25* grew faster, had higher volumes, and weighed more than tumors in the control group (all *P* < 0.05) (Fig. 5A). These results suggested that the *LRP1*–*SNRNP25* fusion might be a novel oncogenic driver of osteosarcoma.

Second, we compared the protein levels of pJNK, MMP2, and Ki-67 between the two groups. The rate of pJNK positivity in osteosarcoma cells with high *LRP1*–*SNRNP25* expression was significantly higher than that in the corresponding control cells (80% vs. 40%), and MMP2 expression was also higher in cells with high *LRP1*–*SNRNP25* expression (100% vs. 30%) (Fig. 5B). These results were consistent with the western blot results at the cellular level, further confirming that *LRP1*–*SNRNP25* promoted the growth of osteosarcoma cells through pJNK and MMP2. In addition, the expression level of Ki-67 was higher than that in the control group.

Third, we evaluated whether the pJNK inhibitor SP600125 inhibits tumor growth in vivo. In nude mice injected with cells overexpressing *LRP1*–*SNRNP25*, the tumor volume and weight were significantly decreased after SP600125 treatment compared with DMSO treatment (*P* < 0.05) (Fig. 5C). In the control group, neither the tumor volume nor the tumor weight changed significantly after SP600125 treatment compared with that in the DMSO group (*P* > 0.05) (Fig. 5D). These results confirm that in vivo, *LRP1*–*SNRNP25* overexpression may promote tumor growth by increasing the pJNK level and that the pJNK inhibitor SP600125 can significantly inhibit tumor growth induced by *LRP1*–*SNRNP25* overexpression.

### *LRP1*–*SNRNP25* promotes tumor metastasis in vivo

**Fig. 6:**
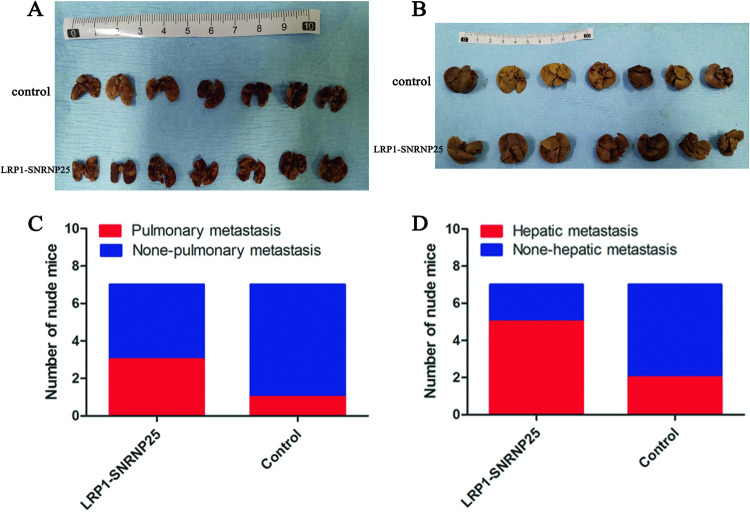
*LRP1*–*SNRNP25* promotes tumor metastasis in vivo. **A** Gross specimen of an excised lung. **B** Gross specimen of an excised liver. **C** Rate of lung metastasis in the *LRP1–SNRNP25* overexpression group and control group. **D** Rate of liver metastasis in the *LRP1–SNRNP25* overexpression group and control group.

In previous experiments at the cellular level, *LRP1*–*SNRNP25* overexpression was found to increase the invasion and migration of osteosarcoma cells. To further confirm this conclusion in vivo, we divided 14 nude mice into an experimental group and a control group, with 7 mice in each group. The experimental group was injected via the tail vein with 143B cells overexpressing *LRP1*–*SNRNP25*, while the control group was injected with empty vector-transfected 143B cells. The nude mice were sacrificed 7 weeks later, and the metastatic nodules in the lungs and livers were counted. Typical lung metastatic nodules are shown in Supplementary Fig. 3. The nude mice injected with cells overexpressing *LRP1*–*SNRNP25* had higher incidences of lung (3/7 mice vs. 1/7 mice) and liver (5/7 mice vs. 2/7 mice) metastasis than the control mice (Fig. 6A–D).

**Fig. 7:**
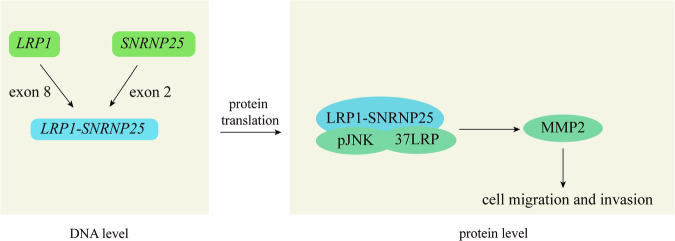
The formation of *LRP1*–*SNRNP25* fusion gene and its role in tumor cell invasion and migration. The *LRP1*–*SNRNP25* fusion gene expression can increase the protein expression and activity levels of pJNK, 37LRP, and MMP2. LRP1–SNRNP25 fusion protein forms a protein complex with pJNK and 37LRP, thus activates MMP2 and plays an important role in promoting the invasion and migration of osteosarcoma cells.

In summary, *LRP1*–*SNRNP25* expression can increase the protein expression and activity levels of pJNK, 37LRP, and MMP2, and LRP1–SNRNP25 fusion protein forms a protein complex with pJNK and 37LRP, thus activates MMP2 and plays important role in promoting the invasion and migration of osteosarcoma cells (Fig. 7). Thus, these data suggest that the fusion gene *LRP1*–*SNRNP25* drives tumor cell invasion and migration by activating the pJNK/37LRP/MMP2 signaling pathway.

## Discussion

Osteosarcoma is a common primary malignant tumor in bone, accounting for approximately 15–20% of primary skeletal tumors [19]. After diagnosis, approximately 50% of patients with osteosarcoma experience some degree of lung metastasis, which is the leading cause of death in patients with osteosarcoma [1, 20]. Over the past 30 years, with pulmonary metastasectomy and neoadjuvant chemotherapy, the cure rate of osteosarcoma has increased; however, the overall survival rate of patients with lung metastasis remains as low as 20% [2]. Importantly, osteosarcoma has a high degree of genomic instability. Therefore, studying the mechanism of osteosarcoma development at the gene level has become an important direction in osteosarcoma research. In this study, based on previous research results, we further confirmed the existence and biogenesis mechanism of the fusion gene *LRP1*–*SNRNP25* at the DNA level by WGS. In our in vitro cell experiments, we found that *LRP1*–*SNRNP25* promotes the invasion and migration of osteosarcoma cells through the pJNK/37LRP/MMP2 signaling pathway, which can be inhibited by treatment with the pJNK inhibitor SP600125 or the MMP2 inhibitor marimastat. In vivo, *LRP1*–*SNRNP25* can promote the growth and metastasis of osteosarcoma. Our results open a new avenue for targeted therapy of patients with *LRP1–SNRNP25*-positive osteosarcoma.

Osteosarcoma is considered one of the most dysregulated malignancies with chromosomal and gene copy number alterations due to its high genomic instability [21]. Fusion genes formed due to genomic instability have rarely been reported in osteosarcoma, with only Debelenko et al. reporting the discovery of a single *EWSR1–CREB3L1* fusion gene in a small-cell osteosarcoma and Kang et al. reporting *Rab22a–NeoF1* [12–14]. However, in 2014, we discovered a novel fusion gene, *LRP1*–*SNRNP25*, and have since been conducting related experiments to further confirm its biological function [15]. In the present study, considering that *LRP1*–*SNRNP25* promoted the proliferation and metastasis of osteosarcoma cells, we further found by evaluating the signaling pathways related to the invasion and migration of osteosarcoma cells that the protein levels of pJNK and MMP2 were increased. By co-IP combined with mass spectrometry, we found that LRP1–SNRNP25, pJNK, and 37LRP interacted, and immunofluorescence staining showed that these three proteins were colocalized and that *LRP1*–*SNRNP25* can increase the protein levels of pJNK and 37LRP. After treatment with the JNK inhibitor SP600125, 37LRP and MMP2 expression was decreased. The expression of MMP2 was also decreased after 37LRP was knocked down. Furthermore, the pJNK inhibitor SP600125, the siRNA targeting 37LRP, and the MMP2 inhibitor marimastat significantly decreased the migration and invasion abilities. Collectively, these findings indicate that the *LRP1*–*SNRNP25* fusion gene promotes the invasion and migration of osteosarcoma cells through the pJNK/37LRP/MMP2 signaling pathway. In fact, it has been shown in a variety of tumor cells that activation of JNK may contribute to tumor development, invasion, angiogenesis, and metastasis through the activation of AP-1 transcription factors [22, 23]. In a study of lung adenocarcinoma, Zhou et al. found that JNK regulates 37LRP expression and that 37LRP expression decreased after treatment with the JNK inhibitor SP600125, consistent with our results [24]. 37LRP, as a precursor of 67-kDa laminin receptor (67LR), is involved in protein translation and constitutes an important component of ribosomes. Many studies have linked 37LRP/67LR with tumor metastasis and invasion [25–27]. In addition, clinical studies have shown that 67LR overexpression is associated with the progression and poor prognosis of colorectal cancer, cervical cancer, breast cancer, and uterine adenocarcinoma [28–31]. MMP2, expressed in almost all human tissues and also known as type IV collagenase, plays a key role in invasion and metastasis and is often associated with tumor development. Tumor cells overexpress MMPs or induce their expression in adjacent stromal cells to promote degradation of the basement membrane and invade surrounding tissues. MMPs play a key role in the progression of malignant tumors. Inhibition of MMP activity can significantly decrease the invasion ability of primary and metastatic tumors. Marimastat is a synthetic, nonspecific low-molecular-weight MMP inhibitor that inhibits the activity of MMP-1, -2, -3, -7, and -9 [32]. Our results are consistent with previous studies showing that marimastat significantly reduced the ability of osteosarcoma cells to invade via regeneration of the basement membrane [33].

This study has several limitations. First, the frequency of *LRP1*–*SNRNP25* fusion in patients with osteosarcoma is unknown, and more patients need to be enrolled in studies to determine this rate. Second, pJNK and MMP2 can also promote tumor invasion and migration in other malignancies, and it may thus be necessary to search for *LRP1*–*SNRNP25*-specific downstream signaling molecules. Third, the effect of the MMP2 inhibitor marimastat on the invasion and migration of osteosarcoma cells in mice injected with *LRP1*–*SNRNP25*-overexpressing osteosarcoma cells was not evaluated. Fourth, we did not use an in vivo animal imaging system to dynamically evaluate the growth of primary tumors and lung and liver metastases and did not monitor the growth of tumors in nude mice in real time after administration of the pJNK inhibitor SP600125; thus, a more detailed and intuitive assessment is lacking.

In summary, this study further confirmed the existence and biogenesis mechanism of the previously discovered fusion gene *LRP1*–*SNRNP25* at the DNA level and revealed the mechanism by which *LRP1*–*SNRNP25* promotes the invasion and metastasis of osteosarcoma cells through the pJNK/37LRP/MMP2 signaling pathway in vivo and in vitro. These findings open a new avenue for targeted therapy of osteosarcoma patients.

## Materials and methods

### DNA extraction and WGS

Genomic DNA was extracted from an *LRP1*–*SNRNP25* fusion-positive tissue sample and used for deep WGS. WGS was performed on the HiSeq X Ten platform by Huada Gene, and raw data of (180 Gb/sample) were obtained (HiSeq X Ten: q30 ≥ 75%, sequencing strategy, PE150). The sequencing data were analyzed by the Bioinformatics Institute of Tampere University in Finland.

### Cell lines and cell culture

The human osteosarcoma cell lines SAOS2 and 143B were purchased from the Cell Resource Center (Beijing headquarters). 143B and SAOS2 cells were cultured in DMEM (Invitrogen, Thermo Fisher Scientific, USA) and DMEM:F-12 medium (ATCC, USA), respectively, supplemented with 10% fetal bovine serum (FBS) (Gibco, Thermo Fisher Scientific, USA) at 37 °C with 5% CO_2_.

### Primers, plasmid construction, and generation of stable cell lines

Based on the results of whole-transcriptome sequencing in a previous study, the *LRP1*–*SNRNP25* fusion gene is formed by the fusion of exon 8 of *LRP1* and exon 2 of *SNRNP25*. NCBI was used to search the nucleotide sequence of exons 1–8 of *LRP1* and exons 2–5 of *SNRNP25*, and Primer Premier software was used to design forward and reverse primers for amplification of the target fragment. The nucleotide sequences of the primers for amplification of *LRP1*–*SNRNP25* were as follows: forward: GAGAACGCGTATGCTGACCCCGCCGTTG; reverse: GAGACTCGAGCTTTTG CCTCAGCTTTTTG.

The full-length cDNA encoding *LRP1*–*SNRNP25* was amplified from fusion gene-positive samples, which were obtained during a previous study and preserved in our laboratory. The cDNA encoding *LRP1*–*SNRNP25* was cloned and inserted into the pCMV6-Entry vector (#PS100001, OriGene, USA). Because no antibody specific for the fusion protein LRP1–SNRNP25 is currently available, to facilitate the subsequent detection of *LRP1*–*SNRNP25*, the vector PCMV6-Entry carries a DDK (flag) tag for detection of *LRP1*–*SNRNP25*. The pCMV6-Entry plasmids carrying *LRP1* and *SNRNP25* were purchased from OriGene (#RC218369, #RC200010).

Transient transfection of cells was performed using a Lipofectamine 3000 kit (Invitrogen, USA) according to the manufacturer’s instructions. After 48 h of transfection, 500 μg/ml G418 was added to the cells for further culture until most of the cells in the control group were no longer viable. After 2 weeks of continuous culture, a single-cell clone was obtained and selected for subsequent expansion. Finally, stable cell lines were constructed.

### Western blot (WB) analysis

Stable cell lines were harvested for western blotting. Harvested cells were lysed in radioimmunoprecipitation assay (RIPA) lysis buffer containing a protease inhibitor, DL-dithiothreitol, and a phosphatase inhibitor and were then centrifuged at 13,000 rpm for 10 min at 4 °C. After total cellular protein was extracted, the protein concentration was measured by the bicinchoninic acid (BCA) method. Proteins were separated by sodium dodecyl sulfate-polyacrylamide gel electrophoresis (SDS-PAGE) at 60 V for 30 min, followed by 120 V until the tracking dye had migrated to the required position. After SDS-PAGE, proteins were transferred to a polyvinylidene fluoride (PVDF) membrane by the wet transfer method. The PVDF membrane was blocked in 5% nonfat milk for 1 h at room temperature. Since no antibody specific for the *LRP1*–*SNRNP25* fusion protein (encoded by the new fusion gene *LRP1*–*SNRNP25*) is currently available and the vector used for expression of the *LRP1*–*SNRNP25* fusion gene contains a DDK (flag) tag, an anti-flag tag antibody was used to evaluate the expression of the LRP1–SNRNP25 fusion protein. Then, the membrane was probed with antibodies specific for DDK (CST#2368S, 1:1000), *LRP1* (CST#64099, 1:1000), *SNRNP25* (Cell Signaling, 1:1000), phospho-FAK (CST#3284S, 1:1000), FAK (CST#13009S, 1:1000), phospho-ERK (CST#9101S, 1:1000), ERK (CST#9102S, 1:1000), phospho-STAT3 (Abcam#ab76315, 1:1000), phospho-AKT (CST#9271S, 1:1000), AKT (CST#9272S, 1:1000), Rac1/2/3 (CST#2465S, 1:1000), RhoA (CST#2117S, 1:1000), phospho-JNK (CST#9251S, 1:1000), JNK (CST#9252T, 1:1000), MMP2 (CST#4022S, 1:1000), MMP9 (Abcam#ab38898, 1:1000), and β-actin (Abcam#ab32572, 1:5000). The membrane was incubated overnight at 4 °C. After the membrane was washed with PBST three times, it was incubated with the secondary antibody. A solution containing enhanced chemiluminescence substrate (Thermo Fisher Scientific, Inc.) was added to the PVDF membrane, which was then exposed for band density analysis with GelPro Analyzer (Media Cybernetics, Silver Spring).

Additionally, LRP1 consists of an extracellular segment of 515 kDa and an intracellular segment of 85 kDa, with the measurable part being the intracellular segment of 85 kDa. SNRNP25 has a molecular weight of 25 kDa, and the molecular weight of LRP1–SNRNP25 DDK (flag) tag is 57 kDa. The molecular weight of 37LRP is 37 kDa.

### Wound healing assay

Cells in each group were seeded in 6-well plates for overnight culture (SAOS2 cells, 2 × 10^5^ cells/ml; 143B cells, 3 × 10^5^ cells/ml) and incubated until they reached 90% confluence. A 10 μl sterile pipette tip was used to draw a horizontal line along a UV-sterilized ruler at regular intervals in the wells of the 6-well plate (three horizontal lines per well) and a vertical line was drawn through the center of the horizontal lines for marking. The cells were gently washed twice with PBS, and serum-free medium was added. The 6-well plate was placed under an inverted microscope for imaging, and the initial position of the cells was recorded. The cells were cultured continuously, and the cells were photographed at the same location with an inverted microscope at 24-h intervals (Leica, Germany). Finally, the area of the scratch in each well was observed and quantified.

### Transwell assays

The effects of *LRP1*–*SNRNP25* on the migration and invasion abilities of osteosarcoma cells were determined by Transwell assays with or without Matrigel. A total of 500 μl of FBS-free cell suspension (5 × 10^4^ cells/well) was added to the upper chamber, which contained a membrane with (invasion assay) or without (migration assay) a Matrigel coating. Then, 700 μl of cell culture medium containing 20% FBS was added to the lower chamber. After 24 h of incubation, the upper chamber was removed from the plate and washed with PBS. After cells that did not pass through the upper chamber membrane were removed by wiping with a cotton swab, the cells remaining in the Transwell chamber were fixed and stained with Three-Step Stain Reagent (Thermo Fisher, USA) according to the instructions. The morphology and number of cells in the Transwell chambers were evaluated with an inverted microscope. Images of three random fields of view per well were acquired under a microscope at 10× magnification, the cells were counted, and statistical analysis was then carried out.

### Immunoprecipitation–mass spectrometry (IP–MS) analysis and coimmunoprecipitation (co-IP)

143B cells with or without DDK-tagged *LRP1*–*SNRNP25* fusion expression were lysed in RIPA lysis buffer, and the supernatants were incubated with an anti-DDK antibody and Protein A + G agarose overnight at 4 °C. The immune complexes were separated by SDS‒PAGE. Silver staining of the gel was carried out with a silver staining kit from Beyotime Company. After silver staining, the differentially expressed bands in the two groups were excised from the gel and sent to Beijing Huada Protein Research and Development Company for mass spectrometry analysis. In brief, 10-μl samples were analyzed on a micrOTOF-Q II mass spectrometer (Bruker Daltonics). After the peaks in the obtained mass spectra were marked with the data analysis software, the mass spectrometry data were analyzed using the MASCOT search engine (version 2.3.01).

143B cells with expression of the DDK-tagged LRP1–SNRNP25 fusion, full-length LRP1 and SNRNP25 were lysed in ice-cold IP lysis buffer (Beyotime) and centrifuged for 10 min at 13,000 × *g*. The protein concentrations were determined by the BCA method. After 30 μl of protein lysate from each group was taken as input, the relevant antibodies and Protein A + G agarose were added to the remaining protein lysates. Then, the protein-containing supernatants were incubated overnight at 4 °C with gentle rotation. After centrifugation for 5 min at 2500 rpm and 4 °C, the supernatants were discarded, and the complexes were washed five times with precooled IP lysis buffer. Then, the samples were boiled in 5× loading buffer and analyzed by western blotting.

### Immunofluorescence colocalization analysis

143B cells with expression of the DDK-tagged *LRP1*–*SNRNP25* fusion, full-length *LRP1* and *SNRNP25* were fixed in 4% paraformaldehyde at room temperature for 30 min, permeabilized in 0.5% Triton X-100 for 20 min at room temperature, blocked in 10% goat serum for 30 min, and incubated overnight at 4 °C with primary antibodies (anti-DDK and anti-pJNK antibody mixture, anti-DDK and anti-37LRP antibody mixture, or anti-37LRP and anti-pJNK antibody mixture). The cells were then washed three times in PBST and incubated with the secondary antibody at room temperature for 1 h. Then, nuclei were restained with 4′,6-diamidino-2-phenylindole. The cover glasses were sealed with mounting solution containing an anti-fluorescence quenching agent and were then dried in the dark after labeling. Finally, images were acquired under a fluorescence microscope, and staining was evaluated.

### SP600125 and marimastat treatment

143B cells expressing the DDK-tagged *LRP1*–*SNRNP25* fusion were cultured. When the cells were 80% confluent, the following treatments were carried out: treatment with SP600125 at concentrations of 5, 10, and 15 μM, or treatment with 2 μM marimastat. After 24 h, total protein was extracted and analyzed by western blotting with the same steps described above. We also carried out Transwell assays with the specific steps the same as described above. In these assays, we added 10 μ SP600125 or 2 μ marimastat to the upper chamber in the experimental group and added the same volume of DMSO in the upper chamber in the control group.

### Lentivirus infection and xenograft assay

The LV8N (EF-1aF/mCherry&Puro) shuttle plasmid containing *LRP1*–*SNRNP25* was constructed: OLIGO software was used to design the forward reverse primers for amplification of the target fragment. NotI and BamHI restriction sites and protective bases were added to these forward reverse primers for subcloning of the vector. The primer sequences were as follows: forward: AGGGTTCCAAGCTTAAGCGGCCGCGCCACCATGCTGACCCCGCCGTTGCTCCTGCTG; reverse: ATCAGTAGAGAGTGTCGGATCCTTAAACCTTATCGTC GTCATCCTTGTAATCCA.

The steps of lentivirus packaging and infection were as follows: 293T cells were cotransfected with LV8N (EF-1aF/mCherry&Puro) and packaging plasmids. Progeny virions released from 293T cells were screened, collected, and used to infect 143B osteosarcoma cells. A stable cell line was screened for and maintained in a medium containing 1 μg/ml puromycin.

Female BALB/c nude mice (4–6 weeks of age) were purchased from Jiangsu Jicui Yaokang Biotechnology Co. Ltd. The nude mice were raised in a specific pathogen-free environment (temperature 24 ± 1 °C, relative humidity 55% ± 5%) in the experimental animal center of Tianjin Cancer Hospital. Drinking water, feed, and bedding were sterilized under high pressure and stored in a specially designated animal room.

143B cells with or without *LRP1*–*SNRNP25* fusion expression were subcutaneously transplanted into 20 nude mice. The sizes of the subcutaneous tumors were measured 7 days after cell inoculation. The longest diameter (a) and shortest diameter (b) of each tumor were measured with a Vernier caliper every 3–4 days. The volume of each tumor was calculated according to the following formula: V (mm^3^) = (a × b × b)/2. The tumor growth curve was generated based on the tumor volumes. Five weeks later, all mice were euthanized, and the tumor masses were removed and weighed.

To study the effect of SP600125 on tumors in nude mice, 143B cells with or without *LRP1*–*SNRNP25* fusion expression were subcutaneously transplanted into 20 nude mice. After 4 weeks of inoculation, 100 μl of SP600125 or DMSO was administered separately through intraperitoneal injection. After 7 successive days of injection, the mice were euthanized, and the tumors were weighed.

In addition, we harvested the lung tissues from all mice in the orthotopic models to evaluate the incidence of lung metastasis. The lung tissues were fixed in 4% paraformaldehyde and embedded in five paraffin blocks. Then, the embedded blocks were sliced into serial sections for further hematoxylin and eosin (HE) staining. Lung metastases were evaluated by two experienced pathologists.

In addition, to evaluate the incidence of liver and lung metastasis in the two groups, we injected 143B cells with or without *LRP1*–*SNRNP25* fusion expression into the tail vein and harvested liver and lung tissues from all model mice. These tissues were fixed in 10% paraformaldehyde. Afterward, the mouse lungs and livers were cut along the longest diameter and embedded in paraffin. Then, the embedded blocks were sliced into serial sections, which were stained with HE. The number of metastatic nodules in the livers and lungs was assessed.

### Immunohistochemical (IHC) staining

We collected tumor samples from the xenograft mouse models, embedded the tumor tissues in paraffin, sliced the paraffin blocks into sections, and then carried out IHC analysis to evaluate the protein levels of LRP1–SNRNP25, Ki-67, pJNK, and MMP2 in the tissue samples of the two groups. In brief, the tissue sections were baked in a 60 °C oven for 1 h. Afterward, the sections were dewaxed and rehydrated by successive immersion in the following liquids: xylene I, 30 min; xylene II, 30 min; anhydrous ethanol, 10 min; 95% alcohol, 5 min; 80% alcohol, 5 min; and 70% alcohol, 5 min. After the tissue samples were subjected to antigen retrieval by heating under high pressure, the corresponding specific primary antibodies (mouse anti-human DDK, mouse anti-human Ki-67, rabbit anti-human pJNK, or rabbit anti-human MMP2) were used to stain each tissue sample overnight at 4 °C. Then, the secondary antibody of the corresponding species was added dropwise before incubation at room temperature for 30 min. Then, the sections were stained with DAB solution (Dako, USA), and nuclei were stained with hematoxylin for 5 min. The slices were dehydrated and cleared by successive incubation in 70% alcohol for 5 min, 80% alcohol for 5 min, 95% alcohol for 5 min, anhydrous alcohol I for 10 min, anhydrous alcohol II for 10 min, xylene I for 30 min, and xylene II for 30 min. Finally, we removed the slices, dried the liquid remaining around the tissue samples, sealed the cover glasses with gum, and evaluated the staining under a microscope after the cover glasses were dry.

### Supplementary information

Supplementary Figure 1

Supplementary Figure 2

Supplementary Figure 3

uncropped western blots

uncropped western blots

uncropped western blots

uncropped western blots

uncropped western blots

uncropped western blots

uncropped western blots

uncropped western blots

uncropped western blots

uncropped western blots

uncropped western blots

Supplementary Data 1

## Data Availability

The raw data supporting the conclusions of this article will be made available by the corresponding author.
