# Supplementary figures and images for: The fusion gene LRP1–SNRNP25 drives invasion and migration by activating the pJNK/37LRP/MMP2 signaling pathway in osteosarcoma
Source: Cell Death Discov. 2024 Apr 27;10:198. doi: 10.1038/s41420-024-01962-z (PMC11055890; doi:10.1038/s41420-024-01962-z)

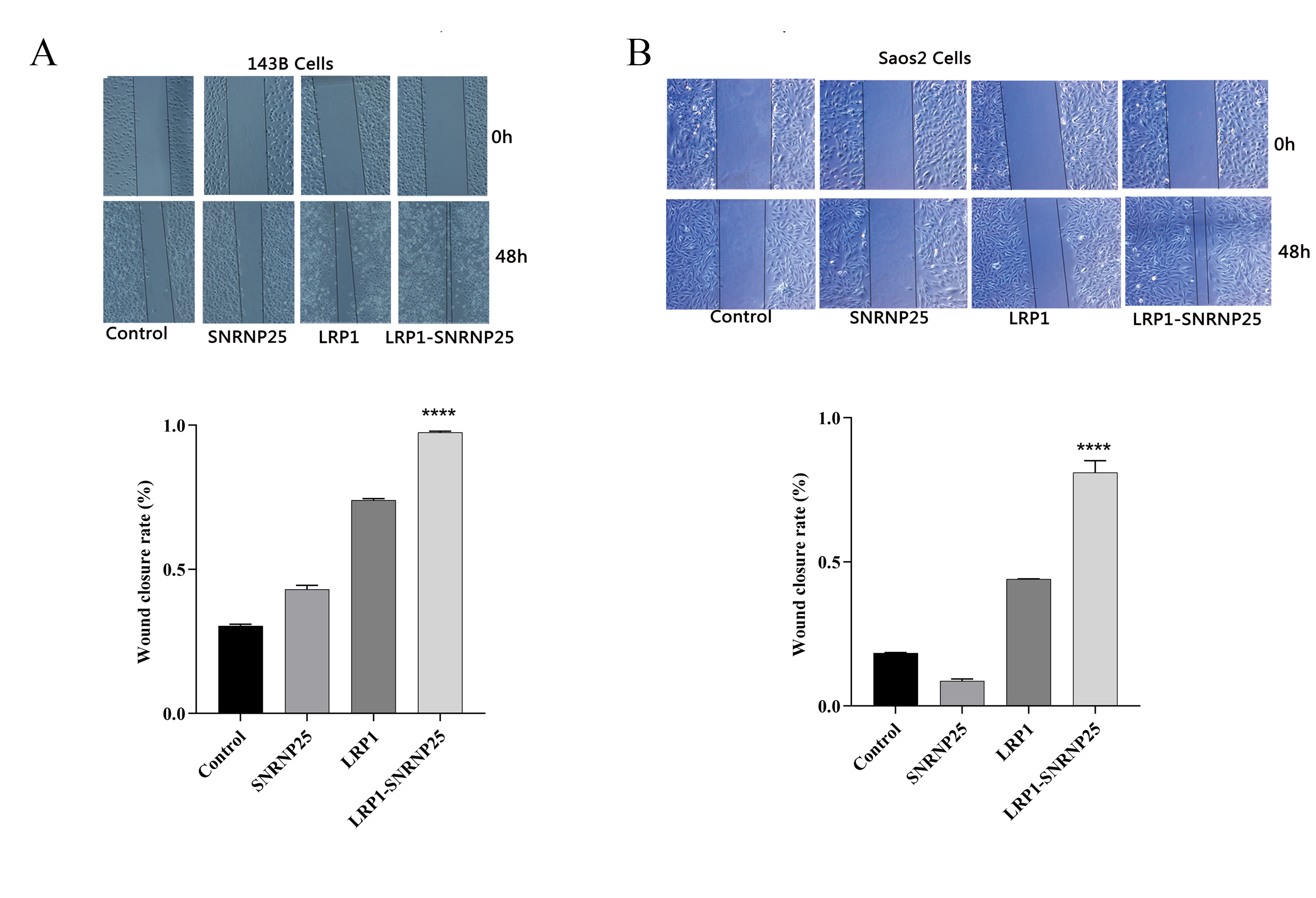

Supplement: Supplementary file 1 — Supplementary Figure 1 [file 41420_2024_1962_MOESM1_ESM.tif]

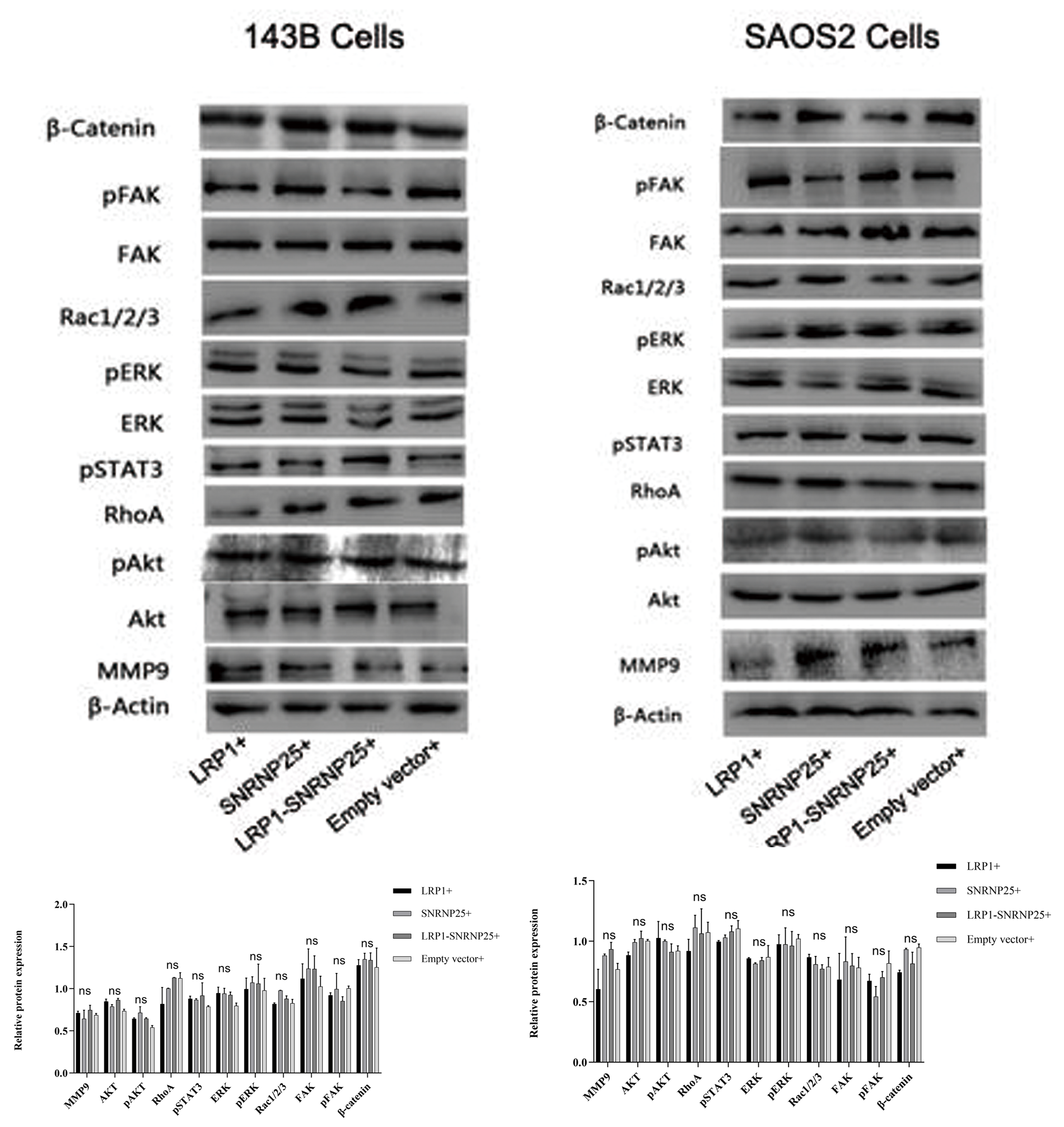

Supplement: Supplementary file 2 — Supplementary Figure 2 [file 41420_2024_1962_MOESM2_ESM.tif]

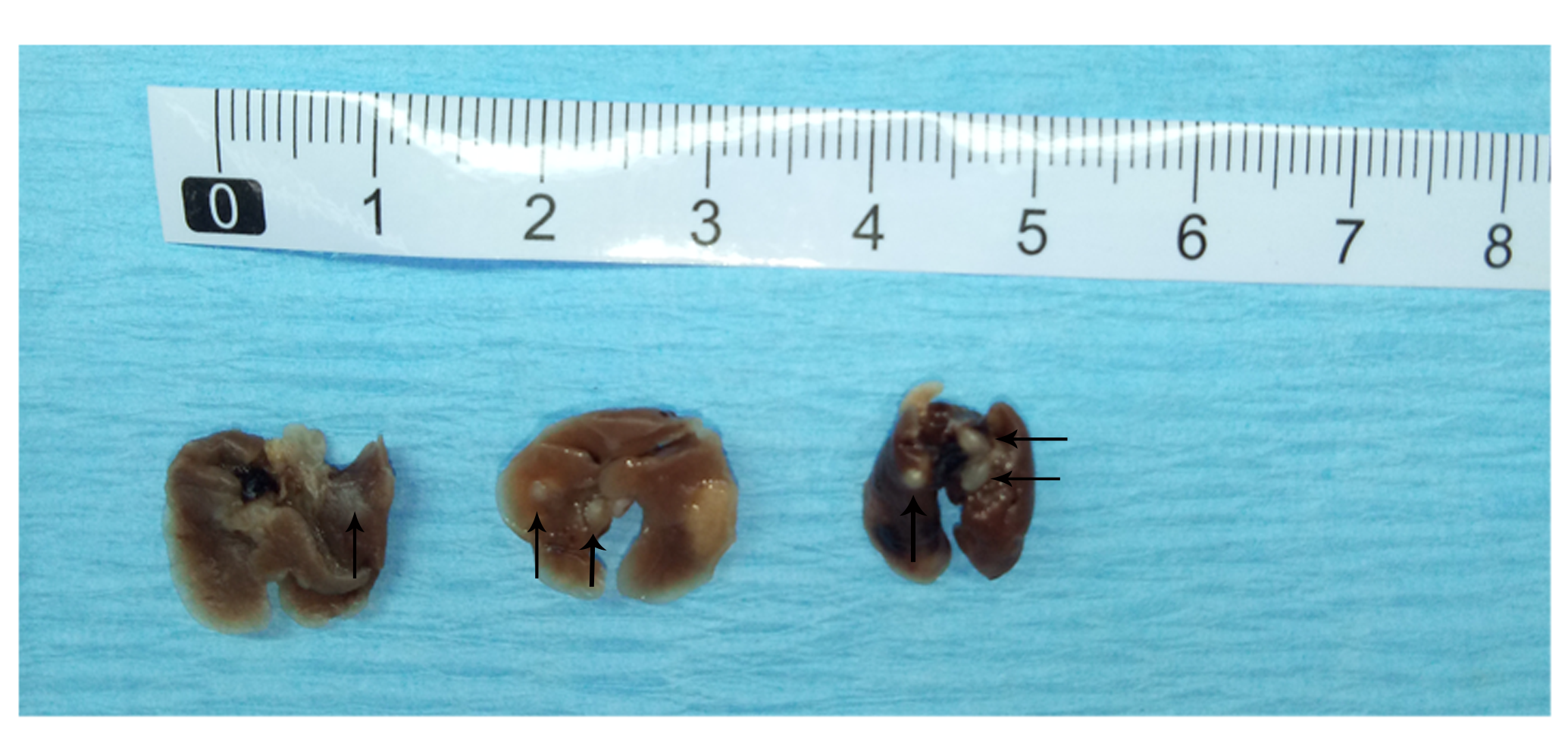

Supplement: Supplementary file 3 — Supplementary Figure 3 [file 41420_2024_1962_MOESM3_ESM.tif]

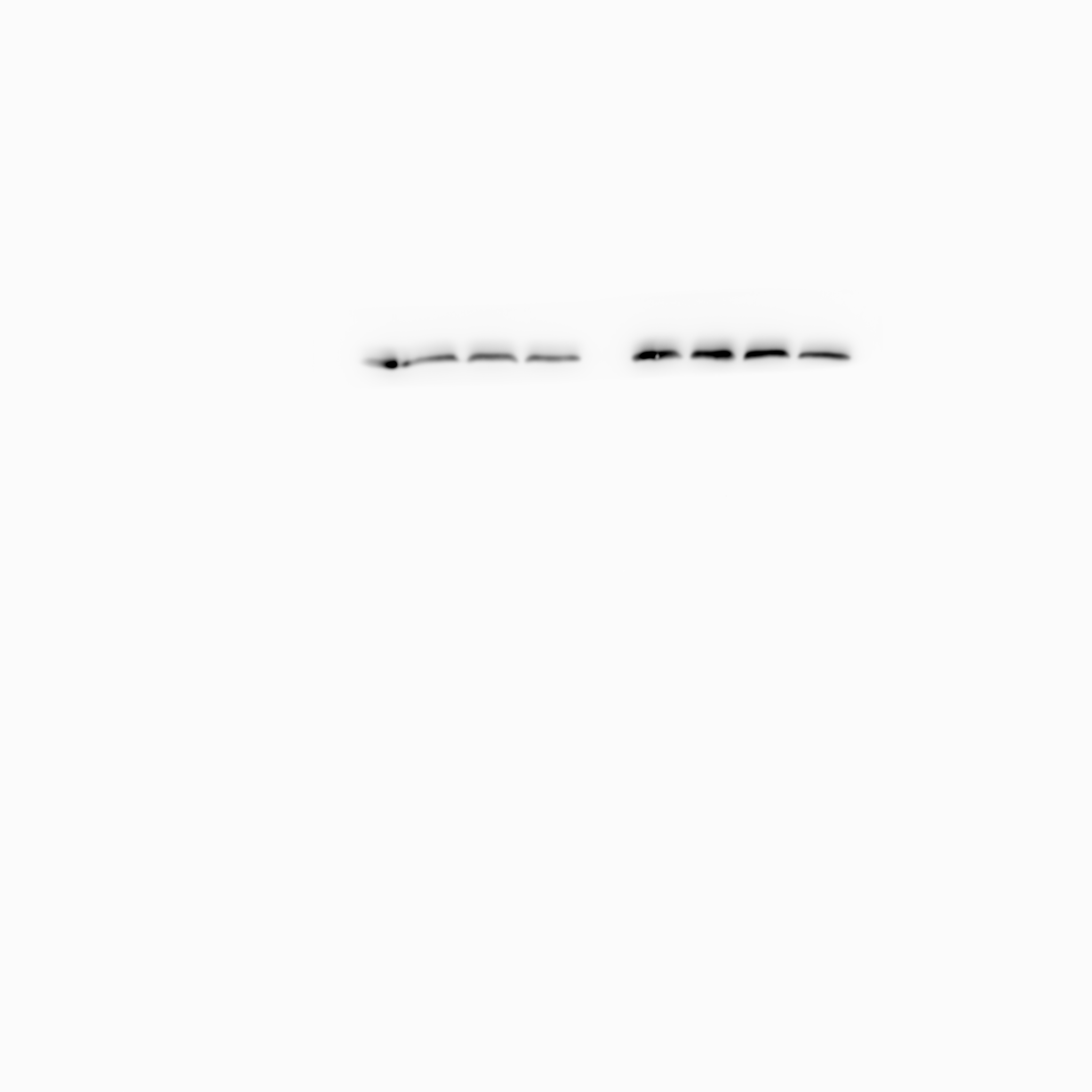

Supplement: Supplementary file 4 — uncropped western blots [file 41420_2024_1962_MOESM4_ESM.tif]

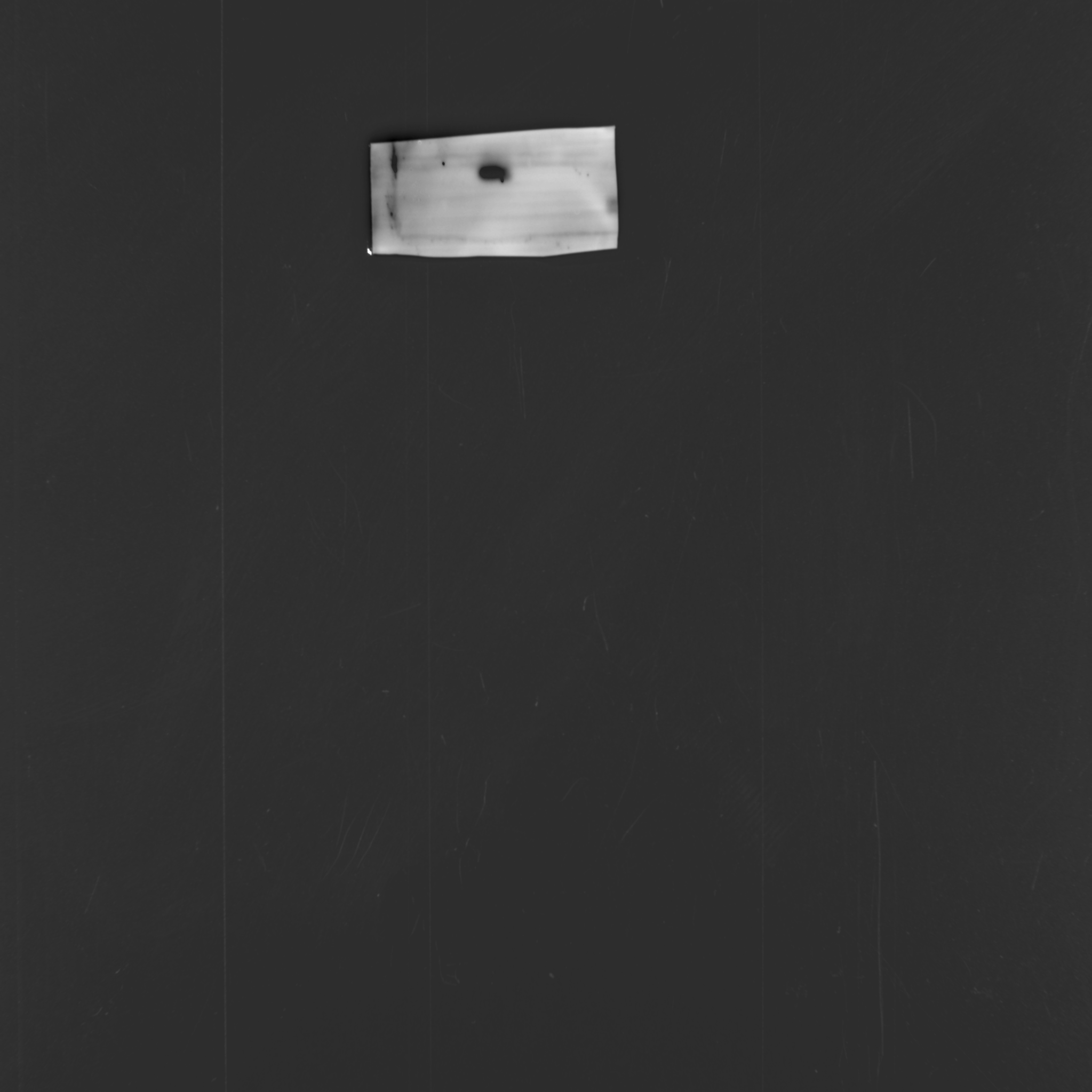

Supplement: Supplementary file 5 — uncropped western blots [file 41420_2024_1962_MOESM5_ESM.tif]

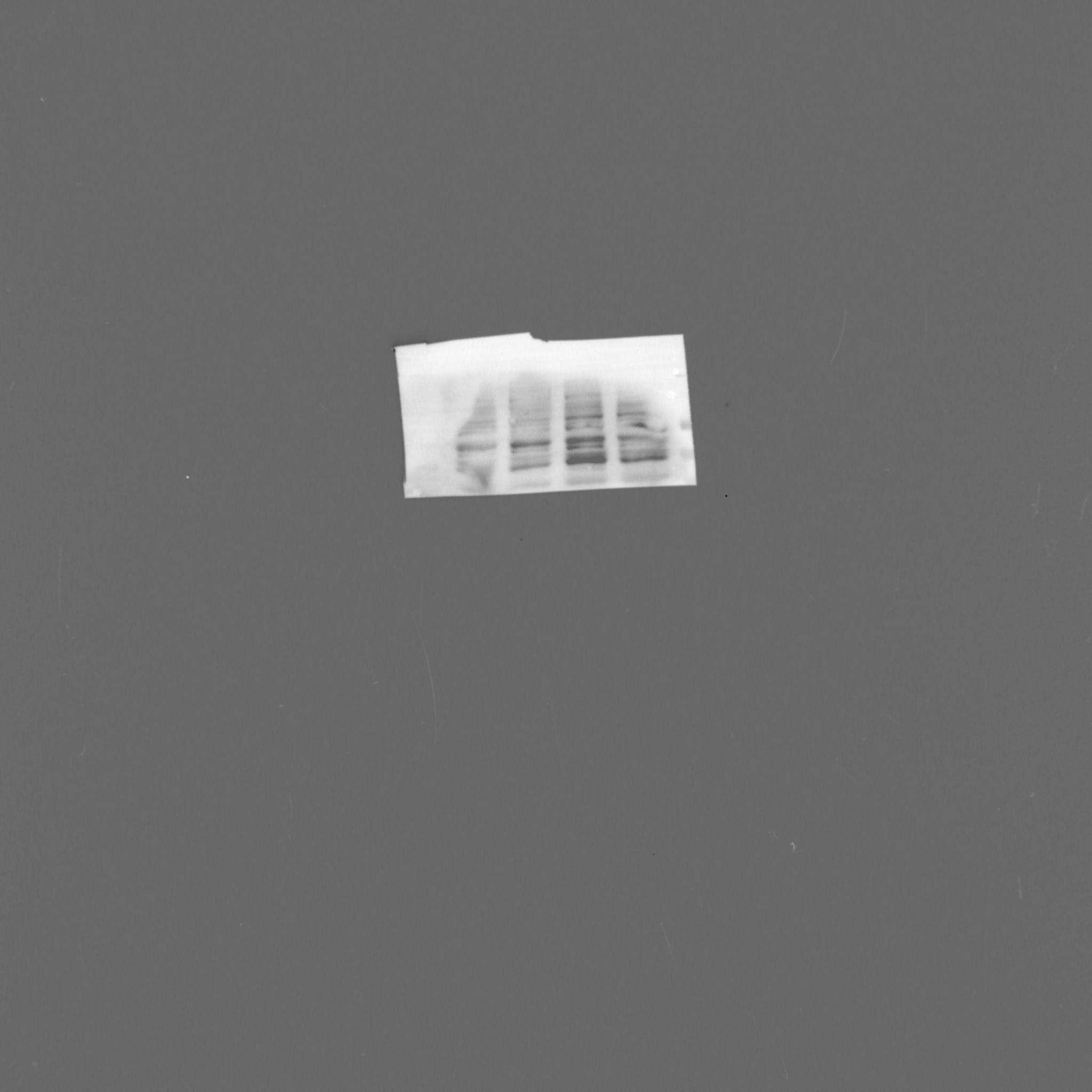

Supplement: Supplementary file 6 — uncropped western blots [file 41420_2024_1962_MOESM6_ESM.tif]

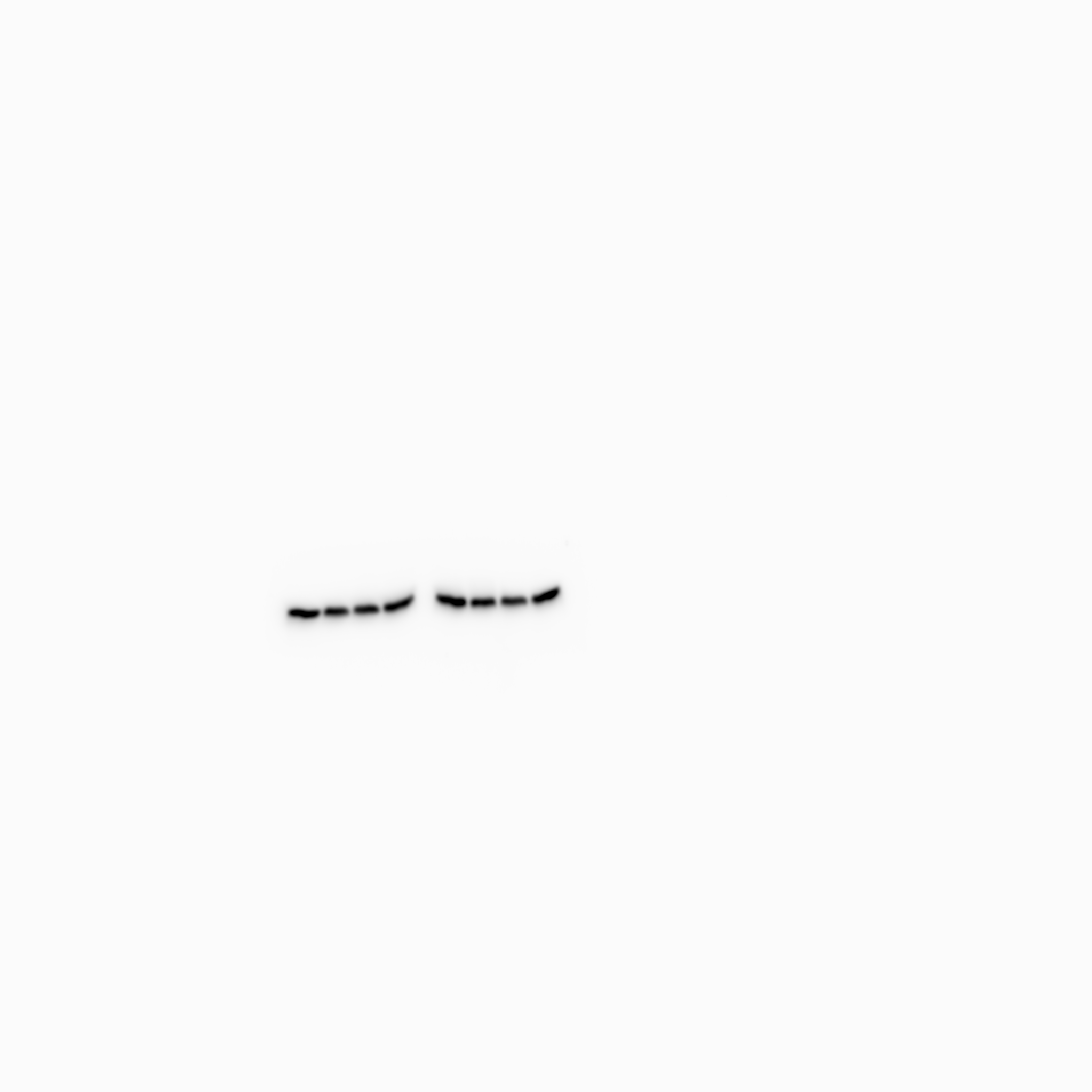

Supplement: Supplementary file 7 — uncropped western blots [file 41420_2024_1962_MOESM7_ESM.tif]

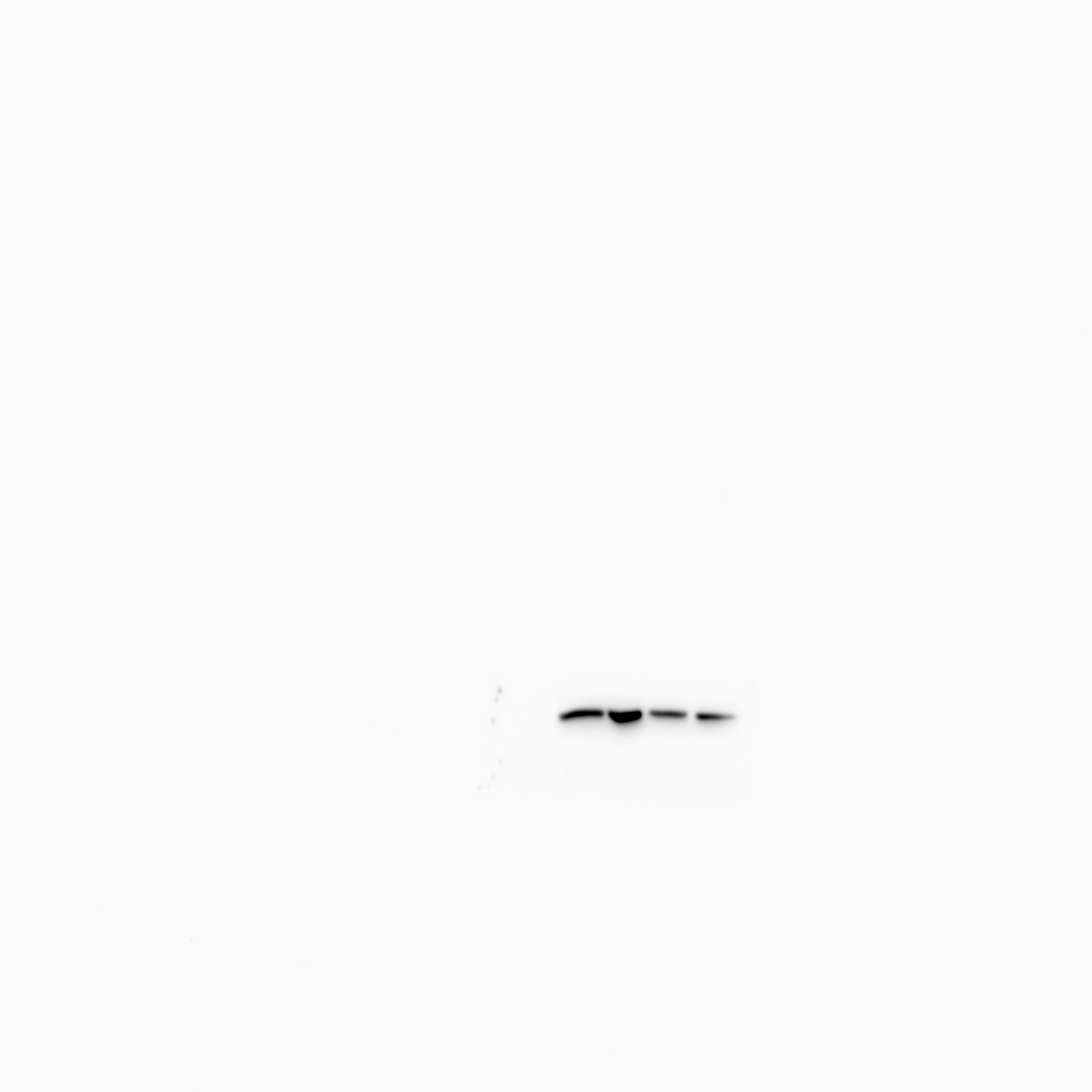

Supplement: Supplementary file 8 — uncropped western blots [file 41420_2024_1962_MOESM8_ESM.tif]

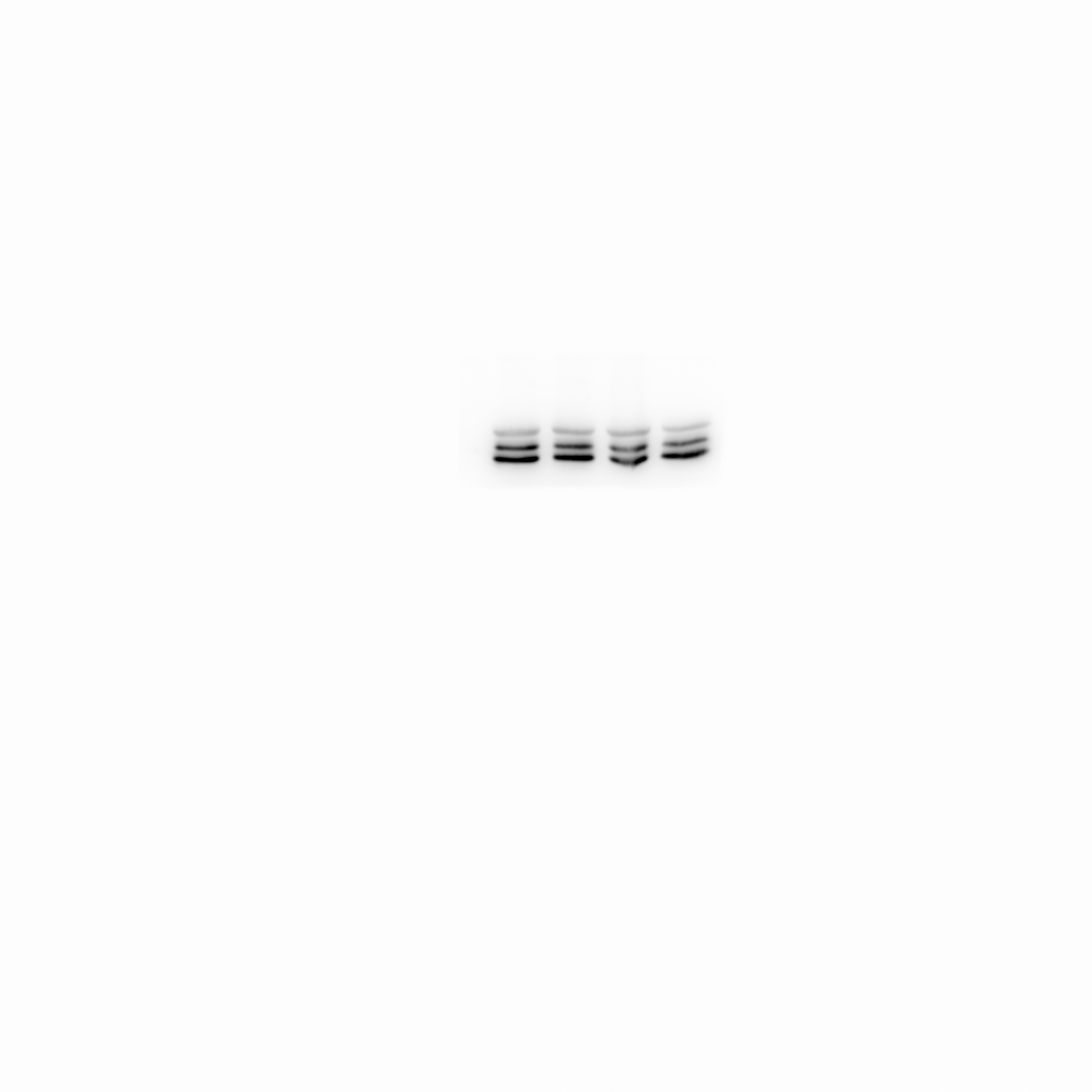

Supplement: Supplementary file 9 — uncropped western blots [file 41420_2024_1962_MOESM9_ESM.tif]

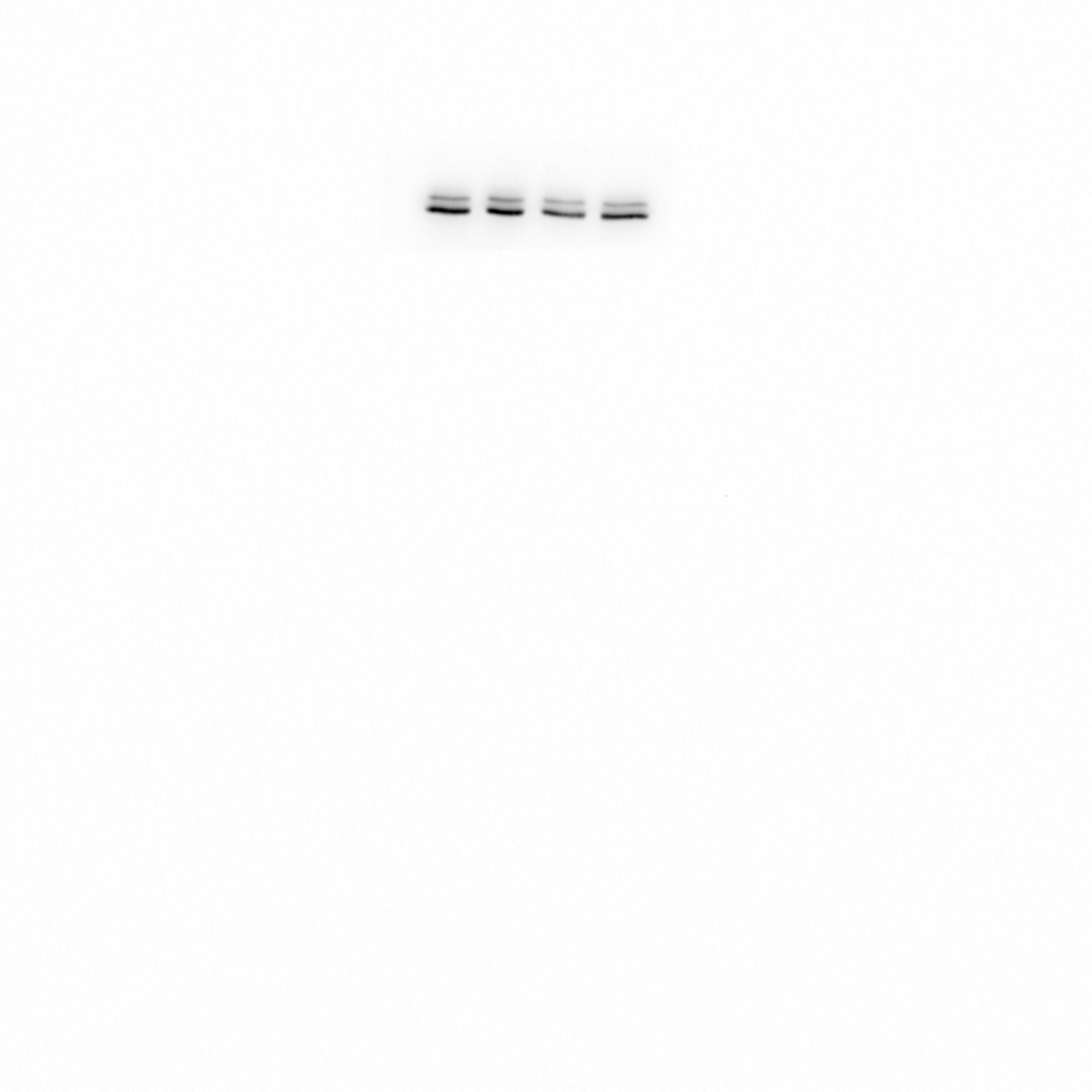

Supplement: Supplementary file 10 — uncropped western blots [file 41420_2024_1962_MOESM10_ESM.tif]

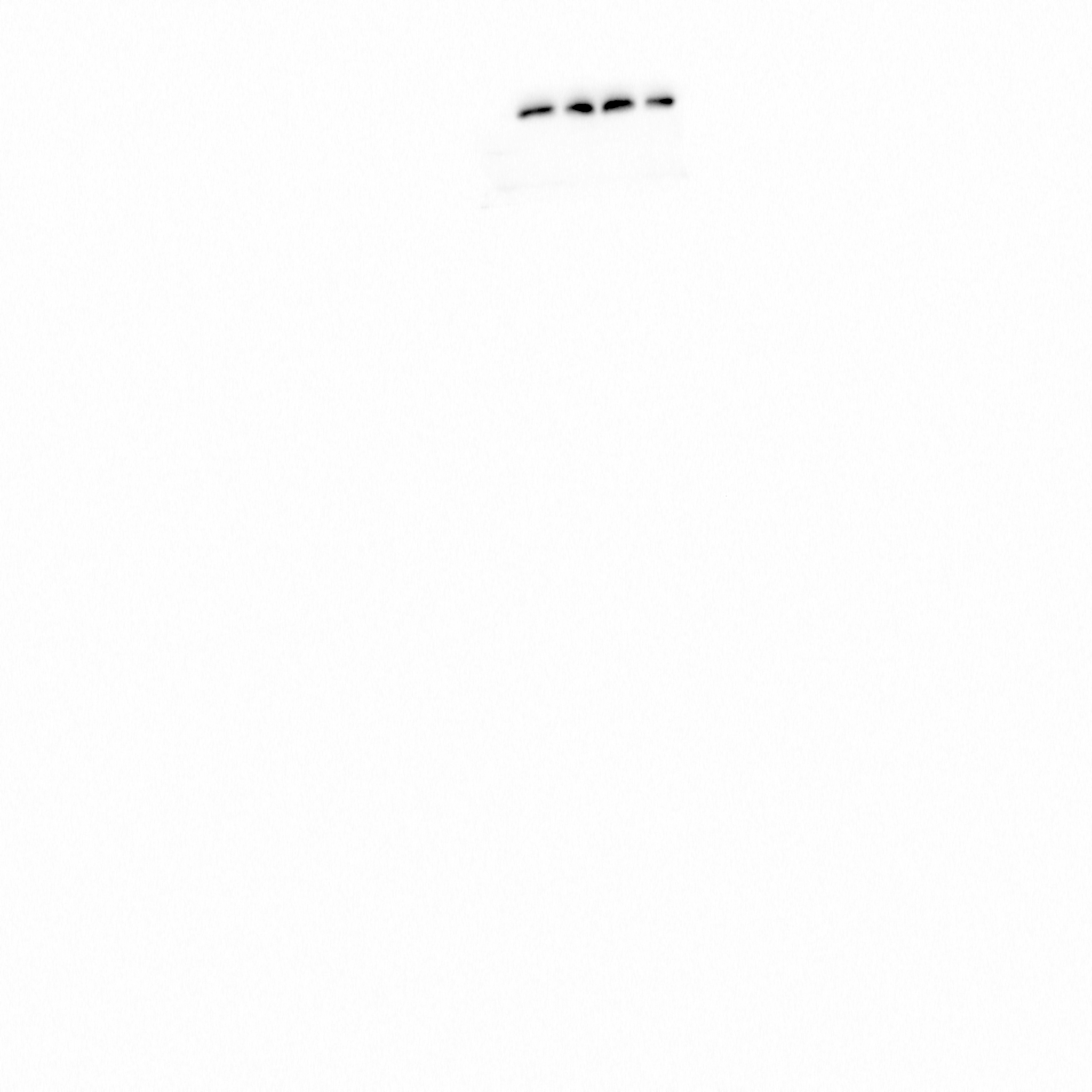

Supplement: Supplementary file 11 — uncropped western blots [file 41420_2024_1962_MOESM11_ESM.tif]

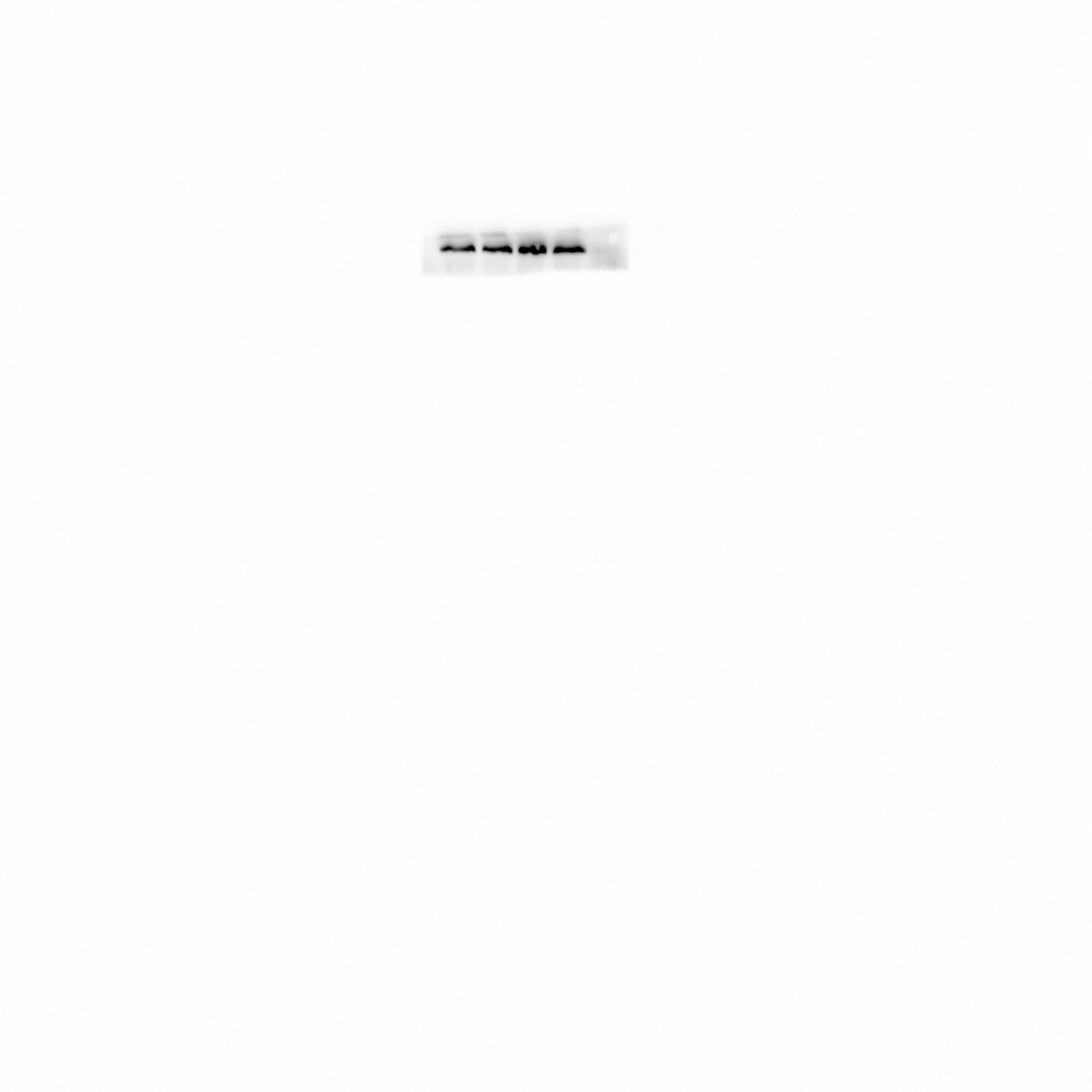

Supplement: Supplementary file 12 — uncropped western blots [file 41420_2024_1962_MOESM12_ESM.tif]

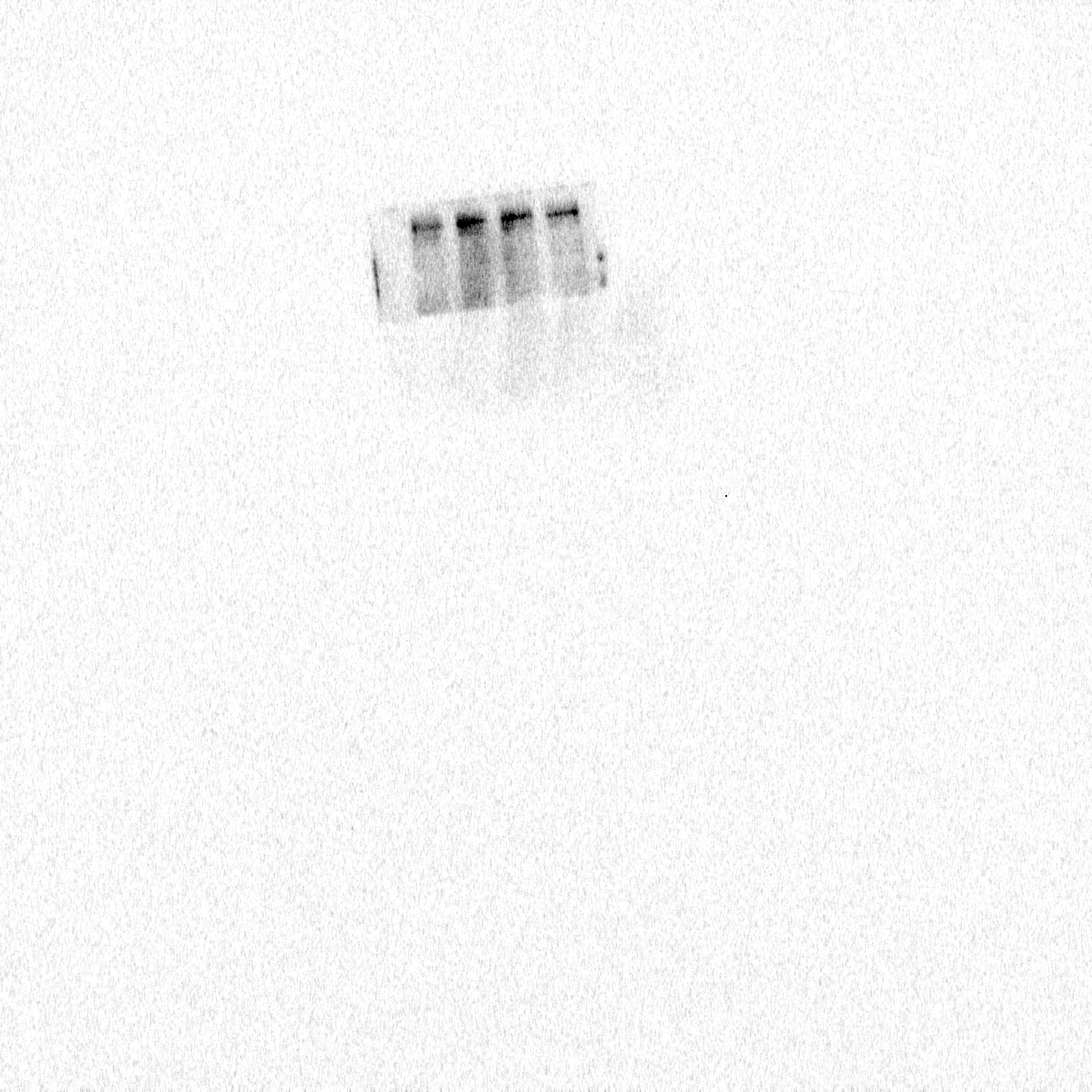

Supplement: Supplementary file 13 — uncropped western blots [file 41420_2024_1962_MOESM13_ESM.tif]

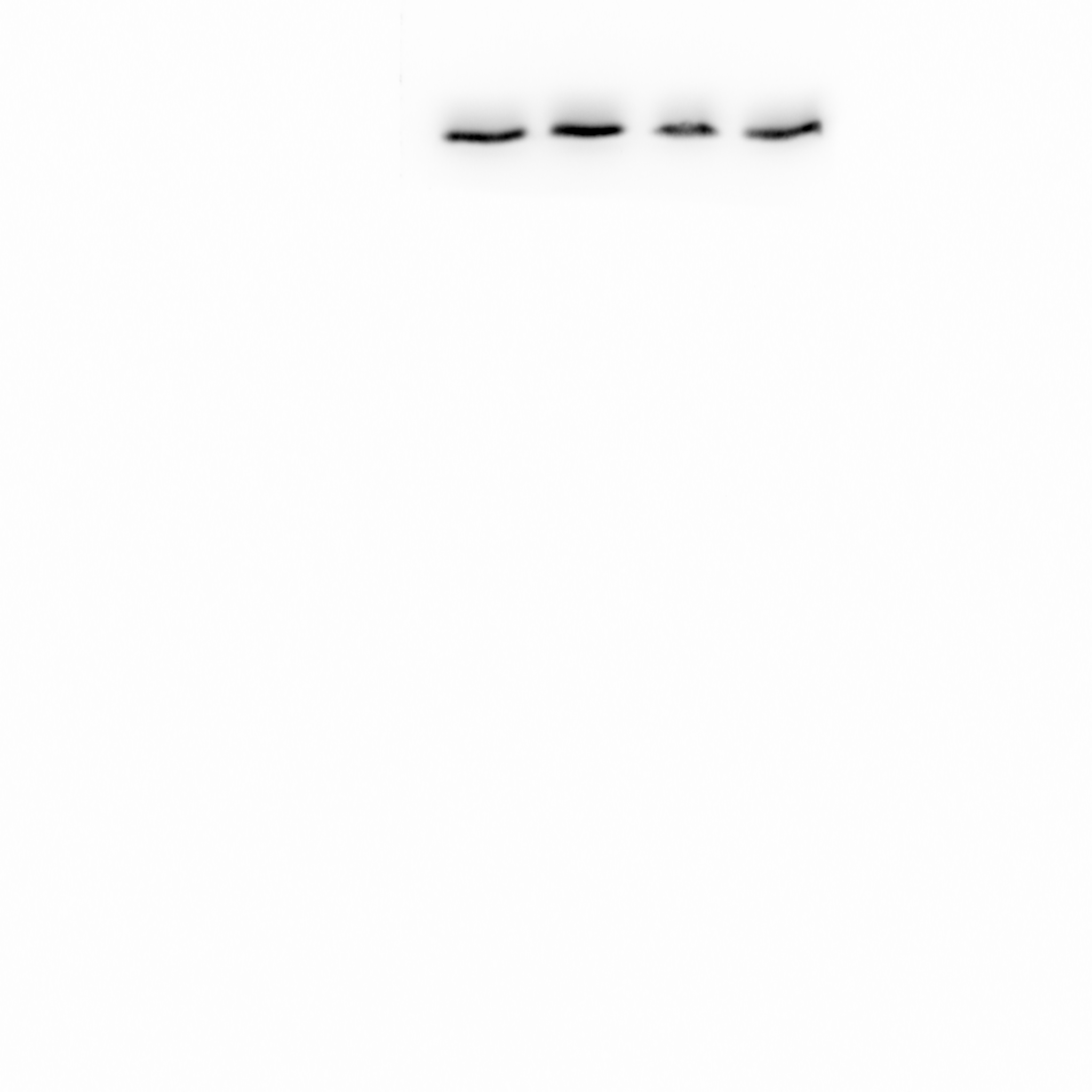

Supplement: Supplementary file 14 — uncropped western blots [file 41420_2024_1962_MOESM14_ESM.tif]
