# Supplementary material for: The fusion gene LRP1–SNRNP25 drives invasion and migration by activating the pJNK/37LRP/MMP2 signaling pathway in osteosarcoma
Source: Cell Death Discov. 2024 Apr 27;10:198. doi: 10.1038/s41420-024-01962-z (PMC11055890; doi:10.1038/s41420-024-01962-z)
Supplement: Supplementary file 15 — Supplementary Data 1 [file 41420_2024_1962_MOESM15_ESM.docx]

**Supplementary Figure 1**: Results of wound healing assay. A: Results of wound healing assay and statistical analysis for the 143B cell line; B: Results of wound healing assay and statistical analysis for the SAOS2 cell line.

**Supplementary Figure 2**: Western blot analysis showed that the protein levels of JNK, ERK/pERK, Akt/pAkt, FAK/pFAK, MMP9, Rac1 and Rho A were not significantly changed in cell lines overexpressing LRP1-SNRNP25.

**Supplementary Figure 3:** Lung metastasis in a mouse (indicated by the arrow).
